# Effect of Lacosamide and Ethosuximide Chronic Treatment on Neural Precursor Cells and Cognitive Functions after Pilocarpine Induced Status Epilepticus in Mice

**DOI:** 10.3390/brainsci11081014

**Published:** 2021-07-30

**Authors:** Aleksandra Szewczyk, Mirosław Zagaja, Joanna Szala-Rycaj, Maciej Maj, Marta Andres-Mach

**Affiliations:** 1 Isobolographic Analysis Laboratory, Institute of Rural Health, Jaczewskiego 2, 20-090 Lublin, Poland; szewczyk.aleksandra@imw.lublin.pl (A.S.); zagaja.miroslaw@imw.lublin.pl (M.Z.); szala-rycaj.joanna@imw.lublin.pl (J.S.-R.); 2 Department of Biopharmacy, Medical University of Lublin, Chodzki 4A, 20-093 Lublin, Poland; ewolucjonista@gmail.com

**Keywords:** pilocarpine, lacosamide, neurogenesis, cognitive functions, ethosuximide

## Abstract

Seizures in about 40% of patients with epilepsy fail to respond to anti-seizure medication (ASM) and may lead to uncontrolled and prolonged seizures often inducing status epilepticus (SE). The aim of the study was to evaluate the impact of a long-term treatment with two different generation ASMs: ethosuximide (ETS, a classic ASM) and lacosamide (LCM, a 3rd generation ASM) on neural stem cells’ (NSCs’) proliferation and learning and memory functions after pilocarpine (PILO)-induced SE in mice. The following drugs were used: LCM (10 mg/kg), ETS (20 mg/kg), and PILO (300 mg/kg). Cell counting was done using confocal microscope and ImageJ software. Cognitive functions were evaluated with the Morris water maze (MWM) test. The level of several selected neurometabolites was measured with magnetic resonance spectroscopy (MRS). Obtained results indicated no significant impact of ETS treatment on the neurogenesis process in PILO mice. Interestingly, LCM significantly decreased the total amount of newborn neurons. The MWM test indicated no significant changes in the time and distance traveled by the ETS and LCM groups compared to PILO control mice, although all measured parameters were more favorable for the PILO mice treated with ASM. Conclusions: The presented results show that long term treatment with LCM and ETS seems to be safe for the cognitive functions and the proper course of neurogenesis in the mouse PILO-induced SE model, although one should remember that LCM administered chronically may act to reduce new neurons’ formation.

## 1. Introduction

Epilepsy is one of the most common neurological diseases. It affects approximately 70 million people worldwide, with the highest incidence observed in children and people over 60 [1,2]. Epilepsy is characterized by a permanent predisposition to generate spontaneous and paroxysmal discharges inside the nerve cells of the cerebral cortex, during which disturbances in consciousness, behavior, motor, sensory, or vegetative functions can be observed [3]. Uncontrolled and prolonged seizures often induce status epilepticus (SE), which may lead to rapid and widespread neuronal damage with incomplete return to the baseline. This state, if not controlled, is very dangerous to the patient’s health and life [4,5].

SE is a particular form of seizure, which lasts longer than a typical seizure or consists of continuous sequential seizures. The most common causes of SE include head injury, febrile seizures, strokes, brain infections, and withdrawal from alcohol and drugs [6]. SE leads to severe respiratory and circulatory disorders and, consequently, to cerebral hypoxia. Prolonged electrical activity in the central nervous system is also associated with neurodegeneration, abnormal neurogenesis in the hippocampus, synaptic reorganization, and behavioral and cognitive deficits, although the extent of these changes depends on the severity and duration of the seizures [6,7]. In the past decades, several animal models of post-SE induced with chemoconvulsants, such as pilocarpine (PILO) or kainic acid (KA), flurothyl, and organophosphates, have been used to generated spontaneous recurrent seizures [8,9]. Studies on PILO or KA models have revealed that some ASMs exert an antiepileptogenic effect [10], which makes them valuable tools in understanding the mechanisms underlying epileptogenesis in TLE. Both models are similar with the human condition regarding the initial brain insult and the electrographical and behavioral abnormalities associated to recurrent pharmacoresistant seizures [11], thus they can be used as the first step in in vivo research evaluating potent anticonvulsant properties of drugs.

Although epileptic seizures have a very negative effect on the functioning of our brain, including cognitive disorders and aberrant neurogenesis, the results from in vitro and in vivo studies confirm that chronic treatment with ASM presents both positive as well as negative influences on the process of apoptosis and neurogenesis [12,13,14]. 

Neurogenesis in the adult brain plays an important role both in maintaining homeostasis and in various disease states of the brain. Epilepsy also has a significant influence on the formation of new cells in the adult brain. It would seem that, as in other neurodegenerative diseases, the loss of existing neurons should be observed; however, the seizure activity in temporal lobe epilepsy (TLE) increases neurogenesis in humans [15], which was also confirmed in animal models of epilepsy [16]. What is more, it has been observed that this type of neurogenesis is not normal (aberrant neurogenesis) and may contribute to the aggravation of the disease. During acute epileptic seizures, there is an increased proliferation of neurons, and the newly formed cells show abnormal migration, morphogenesis, and synaptic integration, creating inappropriate networks of neural connections that initiate and intensify the epileptogenesis process. Thus, increased seizure-induced neurogenesis in adults substantially reorganizes the local nervous system and may impair cognitive functions [17,18]. The abnormal maturation of newly formed neurons may play an important role in the development of chronic seizures. Chronic seizures, in turn, damage and deplete NSCs in the brain, with a consequent reduction in neurogenesis in the advanced stage of epilepsy [19,20]. An extremely important aspect in the treatment of epilepsy is the proper selection of ASMs that will not only stop/prevent seizures but also protect neurons with no negative impact on cognitive functions. 

In the present study, we investigated two ASMs (classified into two various ASM generations) that exhibit anticonvulsant properties working on the thalamus–cortical pathway in impaired awareness as absence in idiopathic generalized epilepsy (ethosuximide, ETS) and focal onset impaired awareness seizure in focal onset seizure (lacosamide, LCM). ETS, a classic ASM, is mainly dedicated to the treatment of epilepsy with primary generalized typical and atypical absences [21]. The main mechanism of action of ETS is the inhibition of T-type low-voltage calcium channels in thalamic nerve cells [22]. In addition, ETS has been shown to partially inactivate sodium channels [23]. LCM is a new generation ASM belonging to the group of functionalized amino acids, which has been proven to possess potent anticonvulsant activity in a broad range of animal models of partial onset and pharmacoresistant seizures, generalized tonic-clonic seizures, and status epilepticus [24,25,26]. The mechanism of action of LCM is based on the slow inactivation of voltage-dependent sodium channels, which leads to the stabilization of the cell membranes of over-excitable neurons [27,28]. In addition, the results of an observational study of 157 adult patients suffering from drug-resistant epilepsy provided information that LCM affects GABA-A receptors and may act synergistically with LEV, improving the GABAergic function [29].

Taking into account the abovementioned mechanism of action for both ASMs as well as different generations of ETS and LCM and the serious neurological consequences of SE in the hippocampus, the main aim of the study was to explore the relationship between long-term treatment with ETS and LCM and hippocampal neurogenesis and cognitive functions in the PILO model of SE in mice.

## 2. Materials and Methods

### 2.1. Animals and Experimental Conditions

All experiments were performed on 6-week-old male C57BL/6J mice (20–22 g). According to the literature data [30] and the results of our recent studies [31,32,33,34], these animals are most suitable for assessing changes in nerve cell proliferation in the brain. The animals were kept by experimental groups in polycarbonate cages in standard laboratory conditions (natural light-dark cycle, temperature 21 ± 1 °C) with constant access to water and feed. After a 7-day acclimatization period and SE induction, the animals were randomly assigned to three experimental groups consisting of eight mice. Experimental procedures related to the care of animals and protocols used in the study were approved by the Local Ethics Committee at the University of Life Science in Lublin (No 35/2016). 

### 2.2. Status Epilepticus (SE) in Mice 

Mice were administered an intraperitoneal (i.p.) injection with a single dose of PILO 300 mg/kg according to the methods described earlier [35]. Mice were carefully observed after PILO injection to catch first symptoms of convulsions. The category and the number of generalized convulsive seizures in each 1/2 h period was tallied. A modified version of the seizure scale described by Racine and coworkers [35] with categories 1–5 was used to identify seizure severity. A mouse that experienced a minimum of 3 generalized convulsive seizure events within 2 h following pilocarpine injection was considered to have undergone status epilepticus (SE). 

### 2.3. Drugs

The following drugs were used in this project: ETS (Sigma, St. Louis, MO, USA), LCM (Vimpat; UCB Pharma, Brussels, Belgium), BrdU (Sigma Aldrich, St. Louis, MO, USA), medetomidine hydrochloride (Tocris Bioscience, Bristol, UK), isoflurane (Baxter, Warsaw, Poland), PILO (MP Biomedicals, LLC, Illkirch-Graffenstaden, France), and methylscopolamine (Sigma Aldrich, St. Louis, MO, USA). All drugs were suspended in a 1% solution of Tween 80 (Sigma, St. Louis, MO, USA) in water for injections (Baxter, Poland) and injected intraperitoneally (i.p.) with 1 mL syringes as a single injection at a constant volume of 10 mL/kg animal body weight.

### 2.4. Drugs Administration

Animals were divided into 3 groups (8 mice per group, *n* = 8):

1. PILO LCM
2. PILO ETS
3. PILO Control group (PILO + 1% solution of Tween 80)

Starting 72 h after SE induction, animals were systematically injected with LCM, ETS, and 1% solution of Tween 80 once a day for a period of 10 days. Fresh drug solutions were prepared ex tempore each day of the experiment and were administered in the following doses: LCM 10 mg/kg and ETS 20 mg/kg. The doses, route of administration and the peaks of action of ASMs were based on data on their pharmacological activity from the literature and our previous studies [33,36]. In addition to the drugs, BrDU (a marker of cell proliferation; 50 mg/kg) was given as one more single injection for the last 5 days of the treatment. Animals were subjected to transcardial perfusion 3 weeks after the last BrdU injection.

### 2.5. Behavioral Study—Spatial Learning and Memory (MWM Test)

Animals underwent a behavioral test 24 h after the last anticonvulsant injection according to the methods described earlier [33,34]. There was one daily session consisting of four 60-s trials (each trial formed a different quadrant of the pool) for five consecutive days. A circular black pool (diameter 120 cm; TSE Systems, Berlin, Germany) was filled with opaque water (24 °C) and mice were trained to swim to a submerged platform in order to escape from the water. First, they were trained with the platform clearly marked by a beacon on the visible platform component (non-spatial training, days 1 and 2) and then they were trained with the beacon removed in the hidden platform component (spatial training, days 3–5). Twenty-four hours after five days of training, the final test (probe test) was performed. Three parameters were measured: escape latency, distance, and time spent in the W-Channel. The swimming patterns of the mice were recorded with a TSE video tracking system VideoMot2 (TSE Systems, Berlin, Germany). Results were analyzed based on the average values of the parameters tested from all quadrants for each animal in the group.

### 2.6. Magnetic Resonance Spectroscopy (MRS)

After performing a behavioral test, five animals from each experimental group were subjected to MRS to evaluate any potent changes in the level of several selected neurometabolites important for the proper process of neurogenesis. The study was carried out by qualified employees of the Center of Experimental Medicine of the Medical University of Lublin using the MRI technique (ClinScan A 7 T animal MRI scanner, Bruker, USA). Two hours prior to imaging, the animal was moved to its cage without food or water. The animals were anesthetized with isoflurane (with 3.0% induction and 1.5–2.0% maintenance). Both animal respiratory rate and body temperature were continuously monitored through the entire experiment with MR-compatible small-animal monitoring system (SA Instruments, Inc., NY, USA). Mice breathed freely during the MR acquisition and the anesthetic concentration was adjusted to maintain the respiratory rate near 40 bpm. Body temperature was maintained at 37 °C with the use of circulating water. 

MR spectra were processed using the Totally Automatic Robust Quantitationin NMR (TARQUIN 4.3.10 version) software, which is dedicated for fully automatic analysis of short echo time in vivo ^1^HMRS. Spectra were analyzed in standard window 0.2 to 4.0 ppm. The unsuppressed water signal measured from the same volume of interest was used as an internal reference for the absolute quantification of metabolites. Selected parameters of neurometabolites were analyzed: NAA, GABA, Glc, Glth, and Gln. A creatinine (Cr) was used as a criterion to quantitatively analyze alterations of the other metabolites [37]. Thus, the ratio of several selected neurometabolite/Cr clinically important for neurogenesis was evaluated: NAA, GABA, Glc, Glth, and Gln.

### 2.7. Brain Slice Preparation

To determine the influence of LCM and ETS on the process of neurogenesis, 3 weeks after the last BrDU injection, mice were anesthetized with isoflurane anesthesia with premedication of medetomidinechydrochloride (0.25 mg/kg), and perfused with ice-cold saline followed by freshly prepared, ice-cold 4% paraformaldehydewith; the procedures are described in our previous studies [31,32,33,34]. The brains were removed and processed, and coronal sections were cut on a vibratome (available in the principal investigator’s workplace (Leica VT1000 S, Wetzlar, Germany) at a thickness of 50 μm for BrdU/NeuN/GFAP staining.

### 2.8. Immunohistochemical Staining

**Figure 1:**
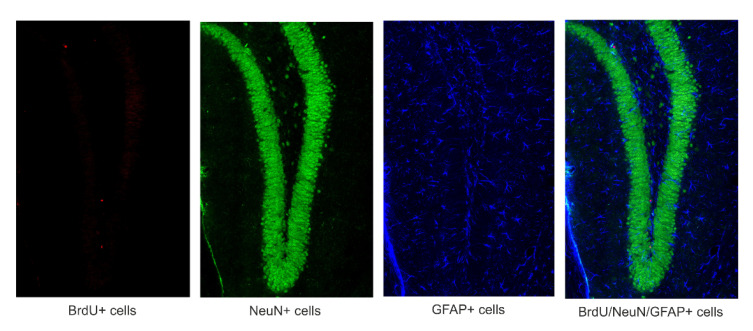
A representative image of BrdU positive cells in colocalization with neurons (NeUN) and astrocytes (GFAP).

In order to evaluate possible changes in proliferation, migration, and differentiation of newly formed cells labeled with BrdU marker into NeuN and GFAP, immunohistochemical staining of BrdU/NeuN and BrdU/GFAP positive cells was performed. Single 50 µm free-floating sections (stored at 4 °C in phosphate-buffered saline PBS with 0.1% sodium azide) were stained according to the methods described previously [31,32,33,34]. A representative image with colocalization of BrdU/NeuN/GFAP positive cells is shown in Figure 1.

### 2.9. Confocal Microscopy and Cell Counting

Confocal imaging was performed using a Nikon A1R confocal system microscope (Tokyo, Japan). Quantitative analysis of total numbers of BrdU+ cells displaying neuron-specific (NeuN) or astrocyte-specific (GFAP) markers were determined using confocal microscopy to score the colocalization of BrdU and phenotypic indicators in the GCL and SGZ of the mouse DG in representative sections from each animal using the methods described in our previous studies [31,32,33,34].

### 2.10. Statistical Analysis of the Results

The results were analyzed using one-way analysis of variance (ANOVA), followed by Dunnett’s test for multiple comparisons, and performed using commercially available GraphPad Prism version 8.0 for Windows (GraphPad Software, San Diego, CA, USA).

## 3. Results

### 3.1. The Impact of Long-Term Treatment with ETS and LCM on the Process of Neurogenesis in the Dentate Subgranular Zone (SGZ) and Granular Cell Layer (GCL) after PILO Induced SE in Mice

**Figure 2:**
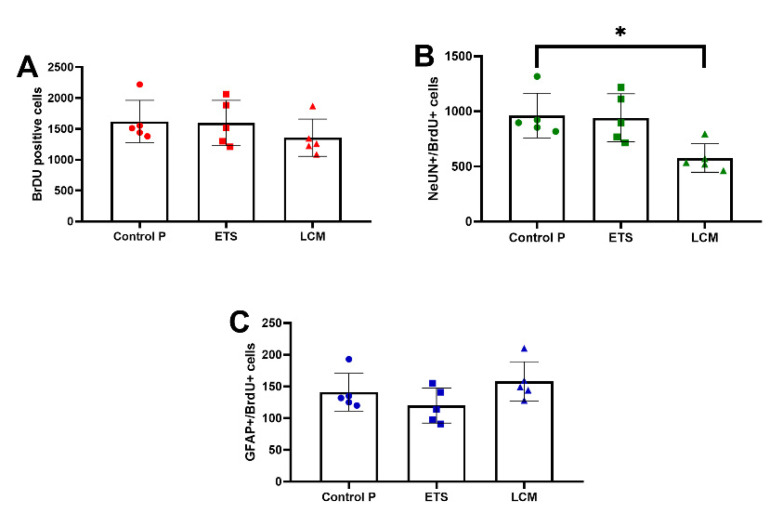
Quantitative analysis of the neurogenesis process after long-term treatment with ETS and LCM in mice after PILO-induced SE. The effect of long-term treatment with ETS and LCM on the total new born cells (**A**), newborn neurons (**B**), and newborn astrocytes (**C**) in the dentate subgranular zone of treated mice. The numbers of cells represent an estimate of the total number of positively labeled cells in the subgranular zone in both hemispheres. The results were analyzed using one-way analysis of variance (ANOVA), followed by Dunnett’s test for multiple comparisons. Each bar represents the mean for five mice; error bars are S.E.M. (* *p* < 0.05, *n* = 5).

Identification of newly formed BrdU+ cells in the SGZ and GCL of the mouse hippocampus was made on the basis of the number of cells that had incorporated the BrdU cell proliferation marker into the DNA. These cells showed positive immunoreactivity with a specific anti-BrdU antibody. Long-term administration of LCM after PILO-induced SE slightly decreased the total number of BrdU-positive cells compared to the PILO control group, but the difference was not statistically significant (1357 ± 135.3 vs. 1621 ± 152.6 respectively, *n* = 5; Figure 2A). ETS did not cause any changes in the BrdU+ cell level compared to the PILO control group (1596 ± 164.6 vs. 1621 ± 152.6 respectively, *n* = 5; Figure 2A).

To study the effects of LCM and ETS treatment on long term survival of newborn neurons, we carried out co-localization of BrdU with neuronal nuclei (NeuN) protein. Immunofluorescence analysis of the number of BrdU/NeuN-co-labeled cells was significantly decreased in LCM-treated mice as compared with the PILO control group. The average number of newborn neurons for LCM PILO mice was 577 ± 57.5 (*p* < 0.05; *n* = 5; Figure 2B) and, for the PILO control group it was 961 ± 90.6 (*p* < 0.05; *n* = 5). No statistically significant changes in NeuN/BrdU+ cell counts were observed in animals receiving chronic ETS (Figure 2B).

Finally, the immunohistochemical staining made it possible to visualize differences in the level of newly formed astrocytes in the hippocampus. These cells are visible thanks to the labeling of the specific glial fibrillary acidic protein (GFAP) contained in them that collocates with BrdU. In the LCM PILO mice, the level of GFAP+ cells was similar to the PILO control group. A slightly decreased level of newborn astrocytes were observed for the ETS PILO group compared to the PILO control group, but the difference was not statistically significant. The mean number of astrocytes for mice receiving LCM was 158 ± 13.93 and for ETS 119.8 ± 12.3, while for the PILO control mice it was 141 ± 13.26 (*n* = 5; Figure 2C).

### 3.2. The Impact of Long-Term Treatment with ETS and LCM on Mouse Spatial Learning and Memory after PILO Induced SE

In order to identify possible disorders of the learning and memory process, the animals were subjected to the Morris water maze test. Three selected parameters were analyzed: (1) the average time needed to find the platform from individual quadrants, (2) the average distance traveled in order to find the platform from each quadrant, and (3) the mean percent of time spent in the W-Channel from all quadrants.

#### 3.2.1. Escape Latency

**Figure 3:**
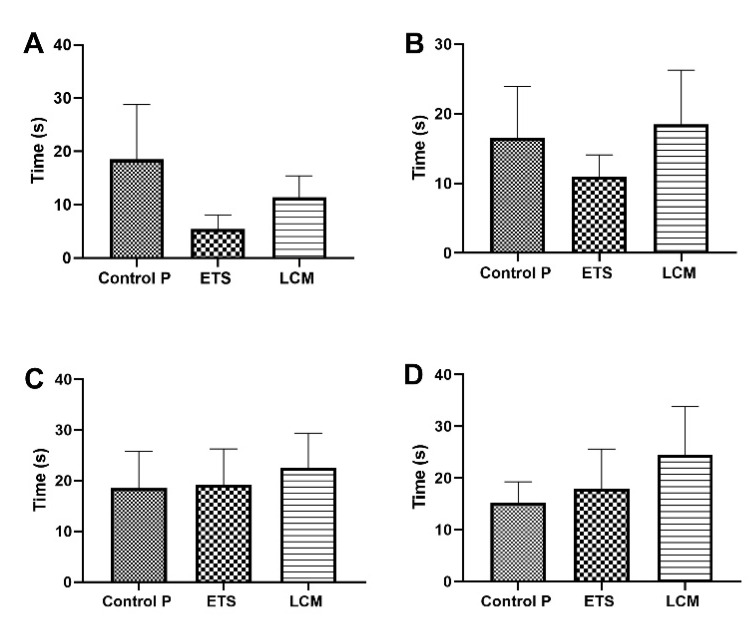
The impact of long-term treatment with ETS and LCM on escape latency after PILO induced SE in mice.(**A**) Target quadrant IQ; (**B**) Left quadrant IIQ; (**C**) Opposite quadrant IIIQ; (**D**) Right quadrant IVQ. The activity was measured on the probe trial of the Morris water maze test. The results were analyzed using one-way analysis of variance (ANOVA) followed by Dunnett’s test for multiple comparisons. Each bar represents the mean for five mice; error bars are S.E.M. (*n* = 8).

Results indicated no statistically significant disturbances in the time needed to find the platform in all treated PILO groups, when compared to the PILO control group (Figure 3). However, the escape latency for LCM mice, starting with the IIQ, IIIQ, and IVQ quadrants, was longer compared to the PILO control group (Figure 3B–D).

#### 3.2.2. Distance

**Figure 4:**
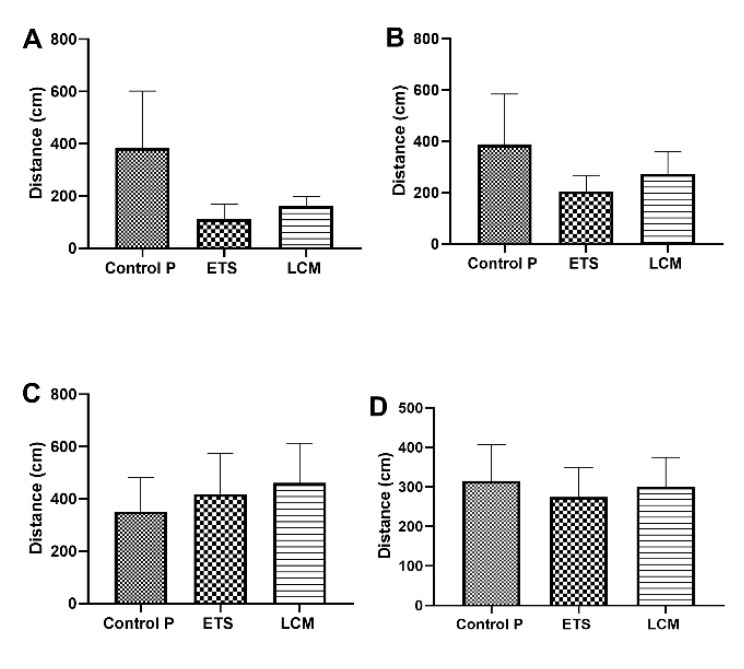
The impact of long-term treatment with ETS and LCM on cumulative distance after PILO induced SE in mice.(**A**) Target quadrant IQ; (**B**) Left quadrant IIQ; (**C**) Opposite quadrant IIIQ; (**D**) Right quadrant IVQ. The activity was measured on the probe trial of the Morris water maze test. The results were analyzed using one-way analysis of variance (ANOVA) followed by Dunnett’s test for multiple comparisons. Each bar represents the mean for five mice; error bars are S.E.M. (*n* = 8).

The obtained results showed that the distance for LCM and ETS-treated PILO mice, starting from the IQ, IIQ, and IVQ quadrant was much shorter than the PILO control group (Figure 4A,B,D), while the distance traveled from the IIIQ quadrant was longer compared to the PILO control mice (Figure 4C). Nevertheless, these differences were not statistically significant.

#### 3.2.3. W-Channel

**Figure 5:**
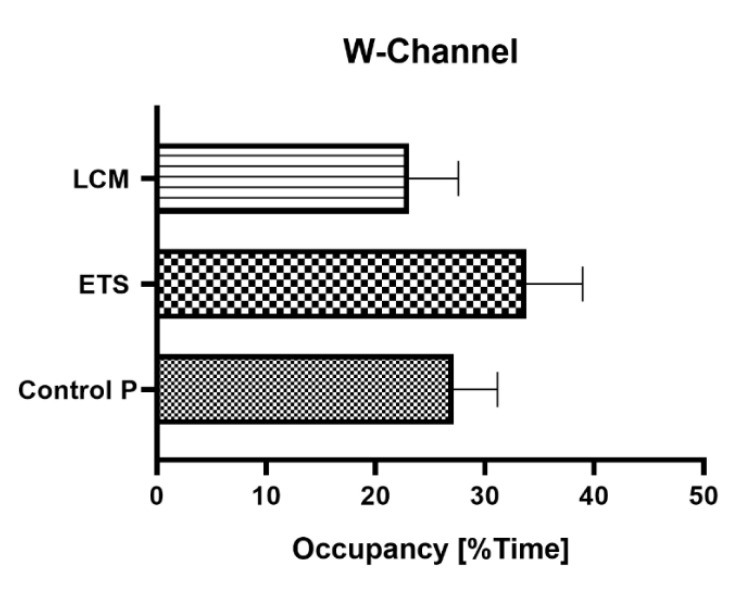
Effect of long-term administration of ETS and LCM on the total average percentage of time spent in the W-Channel.

A very important parameter that enables the tracking of spatial disturbances in the tested animals is the mean percent of time spent in the W-Channel. The averaged values of all quadrants indicated that LCM PILO group spent less time in the channel in comparison to the PILO control mice, while for the ETS PILO group it was higher; however, the differences were not significant (Figure 5).

Five groups of animals were tested and their activity was recorded on the day of the test with a camera placed above the Morris water maze pool. Results were analyzed using one-way analysis of variance (ANOVA), followed by Dunnet’s post-hoc test for multiple comparisons. Each bar represents the mean of eight mice ± standard error of the mean (S.E.M., marked by vertical segments) (*n* = 8).

### 3.3. The Impact of Long-Term Treatment with ETS and LCM on the Level of NAA/Cr, GABA/Cr, Glc/Cr, Glt/Cr, and Gln/Cr in Mouse Brain after PILO-Induced SE

**Figure 6:**
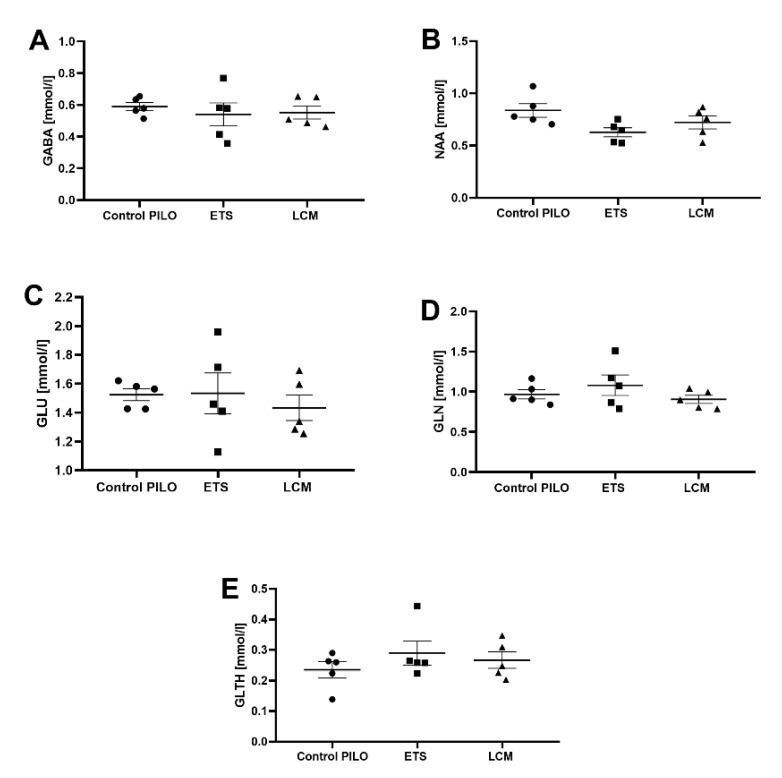
Evaluation of the long-term LCM and ETS administration on the level of (**A**) GABA; (**B**) NAA; (**C**) GLU; (**D**) GLN and (**E**) GLTH in mice after PILO induced SE.

The results obtained from magnetic resonance spectroscopy (MRS) showed no significant changes in the level of metabolites NAA/Cr, GABA/Cr, Glc/Cr, Glth/Cr, and Gln/Cr in all treated PILO groups (Figure 6).

In vivo assessment of neurometabolite levels was performed using a 7T MRI scanner. Results were analyzed using one-way analysis of variance (ANOVA) followed by Dunnet’s post-hoc test for multiple comparisons. Each bar represents the mean of five mice ± standard error of the mean (S.E.M.; *n* = 5). Abbreviations: NAA—N-acetyl aspartate, GABA—gamma-aminobutyric acid, GLU—glucose, GLTH—glutathione, GLN—glutamine. 

## 4. Discussion

The purpose of this study was to evaluate the effects of long-term ETS and LCM treatment on hippocampal neurogenesis and cognitive functions in the PILO model of SE in mice. Research results indicate that long-term administration of ETS does not cause any disturbances in the process of neural stem cells proliferation of treated animals. In contrast, LCM caused a statistically significant decrease in the number of NeuN-positive cells compared to the PILO control group. Results from MRI spectroscopy revealed no statistically significant differences in the level of selected neurometabolites important for the proper course of the neurogenesis process in ETS and LCM mice when compared to the PILO control group. Both ASMs used chronically had no negative impact on learning and memory processes in mice after PILO SE in mice.

Results from our previous studies also indicate that LCM (10 mg/kg) significantly reduces the total amount of newborn cells, including astrocytes, in healthy mouse brain compared to the control group [33]. In turn, Licko et al. [38] showed that 24-day treatment with LCM (10 and 30 mg/kg) in rats after induction of epileptic state with current using intracerebral electrodes (pulse frequency 50 Hz, intensity 700 μA) protected against neuronal cells loss and changes in hippocampal neurogenesis. Similarly to our results, Wang et al. [39] showed that chronic administration of ETS (25 mg/kg and 50 mg/kg) did not affect the number of newly formed neurons and astrocytes, but also did not reduce the frequency of spontaneous seizures when compared to the control group in a lithium PILO model of epilepsy in rats. On the other hand, Tiwari et al. [40] showed that ETS possibly acting through PI3K/AKT/Wnt/β-catenin signaling pathway induces neurogenesis in the rat model of AD-like phenotypes and increases proliferation and neuronal differentiation of in vitro NSCs derived from the rat hippocampus. It should be noted that little information is available regarding the effects of ASM on neurogenesis and these results vary depending on the planned research and the animal model used.

The presented studies assessed the effect of long-term administration of ETS and LCM on the level of selected neurometabolites, showing no changes in the level of tested metabolites in both groups compared to the PILO control group. MRS spectroscopic examination enables the identification and measuring of the levels of neurometabolites and neurotransmitters, which may play an important role in the proper neurogenesis process, such as NAA and Glth, Glu, Gln, and GABA [41]. This method is also gaining popularity in studies evaluating the mechanisms of drug-induced neuronal damage as well as further behavioral and cognitive changes. Moreover, apart from simply identifying brain changes resulting from drug use, MRS has the potential to track disease progression and/or treatment [42]. Currently, it is known that drugs whose mechanism of action is based on the intensification of GABAergic neurotransmission, such as vigabatrin [43], topiramate [44], and gabapentin [44,45], increase the concentration of GABA in the brain. Indirectly, ASM may also affect the concentrations of other metabolites. There is a lot of scientific data from animal models of epilepsy (especially after SE induction) demonstrating a significant decrease in NAA levels [41,46,47]. The NAA level reflects the quantity and functional metabolic state of neurons, and its concentration decreases with neuronal damage and/or dysfunction [48,49]. Moreover, Campos et al. [50] studying TLE patients showed decreased NAA concentrations. On the other hand, a reduced concentration of myo-inositol (Ins) after VPA treatment in patients with epilepsy was observed, while NAA and creatine levels did not change. However, the lower level of Ins was probably not linked with the anti-seizures properties of VPA [51].

In this study, the Morris water maze (MWM) test was performed to assess the cognitive functions of tested mice. The findings of the present study showed that LCM and ETS did not significantly improve the PILO animal’s ability to orientate in space as well as learning and memory skills compared to the PILO control group, although for LCM PILO mice, both parameters, escape latency and distance, were worse in comparison to the ETS treated PILO group. Furthermore, LCM PILO mice spent less time in the direct swim channel, called the W-Channel, which clearly indicated a worse spatial orientation in the tested animals, in comparison to the ETS PILO mice. LCM is a new ASM, and as of today, little information is available about the side effects of this drug in different cognitive domains. However, Nirwan et al. [52] reported that LCM improved PILO-related spatial memory impairment. However, the latest research by Shishmanova-Doseva et al. [53] provided information that LCM has a different effect on learning and memory in healthy and PILO-treated rats. Long-term treatment with LCM, depending on the dose used, leads to cognitive deficits in healthy rats [53,54]. Interestingly, recently we found that chronic administration of LCM causes a negative effect on cognitive functions in the MWM test in healthy mice compared to the control animals [33]. One of the possible mechanisms inducing this effect is the reduction by LCM of BDNF expression and its tropomyosin receptor kinase B (TrkB) receptor in the hippocampus [54]. Treatment with LCM has been shown to be effective in restoring rats’ learning and memory abilities after PTZ-induced epilepsy [55]. It is also worth mentioning studies by Meador et al. [56] which showed that healthy adult patients experienced less cognitive impairment during LCM treatment compared to carbamazepine treatment. Likewise, clinical trials in patients undergoing adjuvant LCM therapy have demonstrated its beneficial cognitive effects [57].

Similarly to our previous results [33] and present study, Churchill et al. [58] showed no negative effect of ETS administered chronically at a dose of 35 mg/kg on the cognitive functions of the adult rats in the MWM test. On the other hand, studies by Ponnusamy and Pradhan [59] proved that chronic administration of ETS in doses of at least 100 mg/kg impaired the performance in the passive avoidance test inducing a disturbance in long-term memory in rats. However, the same rats subjected to the behavioral test using the T-maze test measuring preferences and spatial memory showed no difficulties in performing this task, suggesting that ETS did not negatively affect spatial memory [59]. Moreover, studies by Tiwari et al. [40] found that ETS reverses amyloid-induced β (Aβ) deficits in learning and memory in a rat model of Alzheimer’s disease-like phenotypes by activation of the PI3K/Akt/Wnt/β-catenin pathway.

## 5. Conclusions

In recent years, many researchers have focused their efforts on looking for a single comprehensive ASM capable of not only stopping seizures but also protecting neurons against degeneration and thus cognitive impairment. The obtained results indicate that LCM and ETS seem to be safe from the point of view of cognitive functions and the proper course of the proliferation of newborn cells in the mouse PILO-induced SE model, although one should remember that LCM administered chronically may reduce the formation of new neurons. Bearing in mind long-term outcomes of SE such as subsequent epilepsy, recurrence of SE, cognitive decline, and even mortality, it seems to be of great importance to evaluate already available ASM in terms of their protective properties on neurons and cognitive functions. Beneficial results such as proper neurogenesis, undisturbed learning, and memory functions can certainly qualify these drugs for more advanced pre-clinical investigations, especially in a group of patients suffering from severe SE.

## Data Availability

The data supporting reported results can be found in the laboratory databases of Institute of Rural Health.
